# MetaPalette: a *k*-mer Painting Approach for Metagenomic Taxonomic Profiling and Quantification of Novel Strain Variation

**DOI:** 10.1128/mSystems.00020-16

**Published:** 2016-06-07

**Authors:** David Koslicki, Daniel Falush

**Affiliations:** a Mathematics Department, Oregon State University, Corvallis, Oregon, USA; b Institute of Life Sciences, University of Swansea, Singleton Park, Swansea, United Kingdom; Pacific Northwest National Laboratory

**Keywords:** taxonomic profiling, metagenomics, quantitative methods

## Abstract

Metagenomic profiling is challenging in part because of the highly uneven sampling of the tree of life by genome sequencing projects and the limitations imposed by performing phylogenetic inference at fixed taxonomic ranks. We present the algorithm MetaPalette, which uses long *k*-mer sizes (*k* = 30, 50) to fit a *k*-mer “palette” of a given sample to the *k*-mer palette of reference organisms. By modeling the *k*-mer palettes of unknown organisms, the method also gives an indication of the presence, abundance, and evolutionary relatedness of novel organisms present in the sample. The method returns a traditional, fixed-rank taxonomic profile which is shown on independently simulated data to be one of the most accurate to date. Tree figures are also returned that quantify the relatedness of novel organisms to reference sequences, and the accuracy of such figures is demonstrated on simulated spike-ins and a metagenomic soil sample. The software implementing MetaPalette is available at: https://github.com/dkoslicki/MetaPalette. Pretrained databases are included for *Archaea*, *Bacteria*, *Eukaryota*, and viruses.

**IMPORTANCE** Taxonomic profiling is a challenging first step when analyzing a metagenomic sample. This work presents a method that facilitates fine-scale characterization of the presence, abundance, and evolutionary relatedness of organisms present in a given sample but absent from the training database. We calculate a “*k*-mer palette” which summarizes the information from all reads, not just those in conserved genes or containing taxon-specific markers. The compositions of palettes are easy to model, allowing rapid inference of community composition. In addition to providing strain-level information where applicable, our approach provides taxonomic profiles that are more accurate than those of competing methods.

**Author Video**: An author video summary of this article is available.

## Abstract (precis)

Taxonomic profiling is a challenging first step when analyzing a metagenomic sample. This work presents a method that facilitates fine-scale characterization of the presence, abundance, and evolutionary relatedness of organisms present in a given sample but absent from the training database. We calculate a “*k*-mer palette” which summarizes the information from all reads, not just those in conserved genes or containing taxon-specific markers. The compositions of palettes are easy to model, allowing rapid inference of community composition. In addition to providing strain-level information where applicable, our approach provides taxonomic profiles that are more accurate than those of competing methods.

## INTRODUCTION

Metagenomics is a developing field used to characterize the organismal composition of microbial communities in environmental or clinical samples (1). A key step in most metagenomic analyses is to identify the organisms in the sample and their relative frequencies. A wide variety of different algorithms have been developed for this purpose.

Most approaches, including the one described here, are based on relating sequenced reads to reference organism genome sequences. Conceptually, the aim of these approaches is to place the organisms in the sample on a “tree of life” that has been defined in advance. In practice, the available reference organisms are extremely unevenly scattered through the true tree of life. Many medically important branches, such as enterobacteria, are relatively well sampled, with many strains from the same species, while there are entire phyla of unculturable organisms that are unrepresented (2–5).

A further difficulty, both in theory and in practice, is that a fully resolved tree of life cannot be established, even from complete reference genomes. At the scale of individual species, homologous recombination scrambles variation so that a tree is not necessarily an appropriate representation of organismal relationships, while more distant phylogenetic relationships can be difficult to estimate due to the various technical challenges of reconstructing ancient evolutionary events (6–10).

Based on these practical considerations, an effective metagenomic method should both identify the closest organism or set of organisms in the reference set and also estimate the genetic difference between the closest reference(s) and the organism present in the sample. The method should work both if the closest neighbor is a distant member of the same phylum and if there are multiple strains within the species in question. Fine-scale classification is important because the detailed knowledge we have, of *E. coli*, for example, shows that organisms from the same species can have entirely different ecologies and phenotypic effects on their host (11).

Given these difficulties, a number of different approaches are taken to characterize metagenomic samples. A commonly used approach is to first place individual reads onto a tree constructed for a particular set of genes and then attempt to sum the phylogenetic information across the reads (12–15). Phylogenetic analysis of each read can be computationally challenging for large datasets, and individual reads can often only be placed inaccurately. It is challenging to appropriately represent this uncertainty in later stages of the analysis. These approaches also break down if a tree is not a good representation of relationships among organisms, e.g., within species. Furthermore, while utilizing specific genes (so-called *marker genes*) can increase computational efficiency, this approach throws away a considerable amount of information from sequences that do not align to the marker genes. As a result of these issues, these methods are typically accurate for genus level or higher classification but not for fine-scale classification.

Another approach identifies features that are characteristic of particular organisms, such as the frequency of *k*-mers (16–18). These features are used either for taxonomic binning of individual reads or in order to compute the overall composition. Depending on the *k*-mer size utilized, these methods either are suitable only for higher-level phylogenetic analysis (for small *k*-mers) or are highly dependent on the training database utilized (for larger *k*-mers). In either case, no existing method using this approach can accurately detect and classify organisms that are highly diverged from ones in the training database, and the existing methods still struggle with quantifying strain-level variation. Using longer *k*-mers allows for higher specificity, but using *k*-mers that are unique to specific taxa in the reference data set (as in references 16 and 17) ignores a great deal of information about evolutionary relatedness provided by other *k*-mers. It also makes the approach highly dependent on the specific composition of the reference data set. We argue that utilizing all *k*-mers in a reference database and multiple *k*-mer sizes allows the modeling of the *k*-mer signatures of organisms absent from a given training database.

**FIG 1:**
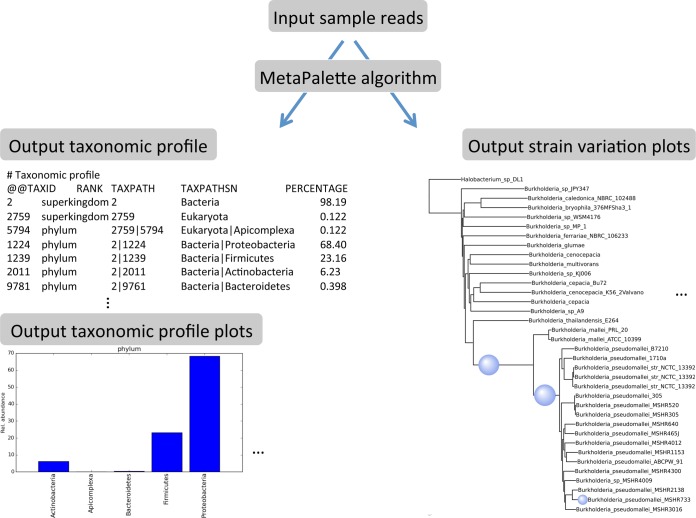
Illustration of the MetaPalette algorithm. Along with an output taxonomic profile and bar chart plots at all inferred taxonomic ranks, figures of strain-level variation for each inferred genus and/or species are also output.

In this paper, we present an approach based on defining a “palette” for each reference organism. Specifically, we count the number of *k*-mers found in the sample DNA that are present in each reference organism. Our approach thus uses all *k*-mers of a particular length in the reference data set, while discarding the specific information provided by matches of individual *k*-mers. This is similar in spirit to the so-called pseudoalignment approach in reference 19, except that here, we use *k*-mer counts of the entire sample, not of individual reads whose origins may be ambiguous. We model these palettes using a simple linear mixture model which includes both the reference organisms and “hypothetical organisms” of different degrees of genetic relatedness to the reference organisms. The algorithm is called MetaPalette, and the outputs of the algorithm are demonstrated in Fig. 1.

We first introduce the concept of a “common *k*-mer matrix” and demonstrate how utilizing multiple *k*-mer sizes allows accurate quantification of evolutionary relatedness. We then develop a mixture modeling procedure that utilizes this information to taxonomically profile a metagenomic sample and indicate the evolutionary relatedness of novel organisms. Evidence on simulated and real data is given that demonstrates that this approach can accurately capture strain-level variation, and we then benchmark this approach against other, commonly utilized metagenomic-profiling techniques.

## RESULTS

### MetaPalette design.

We begin by introducing the basic analytic approach. For a more detailed description, see Materials and Methods. 

### (i) Common *k*-mer training matrix.

To quantify the similarity of two genomes, we count (with multiplicity) the fraction of each genome’s *k*-mers that are in common with the other. Rigorous mathematical definitions of this and other quantities are contained in Materials and Methods. This quantity, denoted for pckm_*k*_(·,·) “percentage of common *k*-mers,” is similar to the well-known Jaccard index (20) except that, among other differences, pckm_*k*_(·,·) is not symmetric but does incorporate the counts of *k*-mers, not just their occurrence.

**FIG 2:**
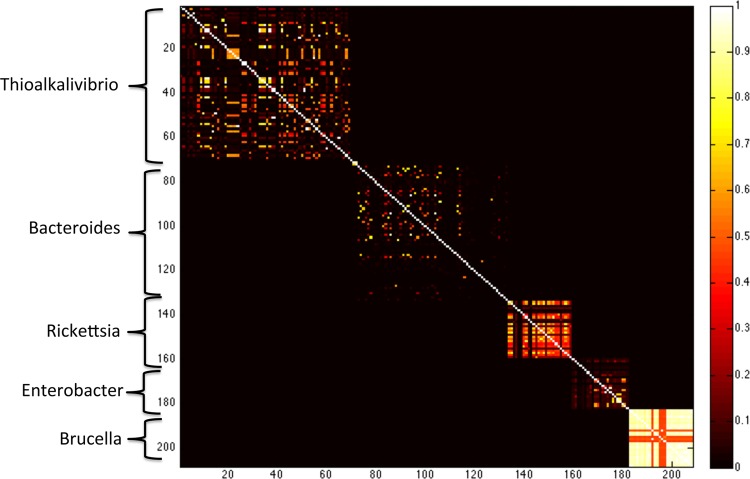
Heatmap of the common *k*-mer matrix *A*^(40)^ for *k* = 40 using a subset of the NCBI bacterial genome database. Delineations between genera can clearly be seen. In a given genus, differing similarities of species are also visible.

When given a set of genomes (i.e., a training database), a pairwise similarity matrix can be formed using the equation *A*_*i,j*_^(*k*)^ = pckm_*k*_(*g_i_,g_j_*) for *g_i_* and *g_j_* training genomes. The column vector pckm_*k*_(·,*g_j_*) can be thought of as a palette, representing the particular *k*-mer profile of *g*_*i*_ in relation to those of other genomes. We call each of these matrices a “common *k*-mer matrix.” These matrices reflect the relatedness of the training genomes based on *k*-mer similarity. For larger *k*-mer sizes, one can clearly extract taxonomic information from these matrices, as shown in Fig. 2.

**FIG 3:**
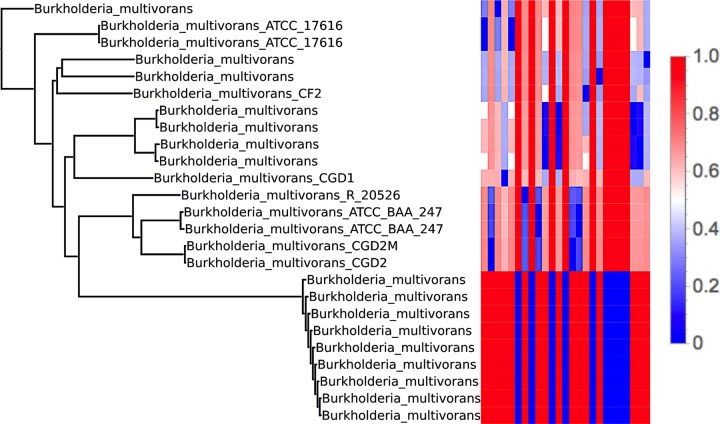
Neighbor-joining tree for the species *Burkholderia multivorans* based on the average of the common 30-mer and 50-mer matrices (shown in heat map to the right), depicting the ability of the common *k*-mer matrices to capture strain-level variation.

Beyond genus-level variation, strain-level variation can be captured through these common *k*-mer matrices. For example, using all the strains of the species *Burkholderia multivorans* accessible via NCBI, we formed a neighbor-joining tree using the average of the 30-mer and 50-mer common *k*-mer matrices. This tree, shown in Fig. 3, demonstrates how the common *k*-mer matrices can capture variations among these strains.

The entries of *A^(k)^* can be calculated in a computationally efficient manner. We take the approach of forming bloom count filters (using Jellyfish [21]) for each of the training genomes and then counting the common *k*-mers using a simple C++ program based on heap data structures.

### (ii) Modeling related organisms.

**FIG 4:**
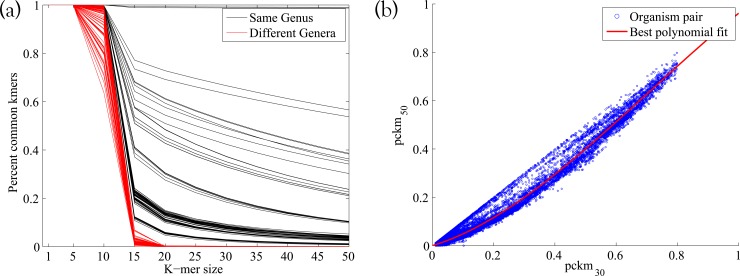
(a) Plot of *k*-mer similarity pckm_*k*_(*g*_*i*_,*g*_*j*_) as a function of *k* for 100 organism pairs of the same genus and 100 of different genera. (b) Scatterplot of the 6,914^2^ pairs of entries of the common 30-mer and 50-mer matrices shown with the best-fit polynomial.

To model the *k*-mer counts for organisms with various degrees of relation from the training database, we take advantage of the differing behavior of pckm_*k*_(·,·) as a function of *k* for closely related organisms and distantly related organisms. In particular, the percentage of common *k*-mers, pckm_*k*_(·,·), decays more slowly as a function of *k* for closely related organisms than for distantly related ones. This is consistent with the intuitive idea that, for example, two organisms from different phyla will have similar percentages of shared 1-mers but very few common 50-mers. Conversely, two closely related strains will have both high percentages of shared 1-mers and high percentages of shared 50-mers. This is demonstrated in Fig. 4a. This property means that using more than one *k*-mer length should in principle allow us to distinguish between having an organism that is identical to a training organism at a low frequency and having an organism that is distantly related to all training organisms but present in the sample at a higher frequency.

We focus on two particular *k*-mer sizes, *k* = 30 and *k* = 50, due to the predictability of pckm_*k*_ for these *k*-mer sizes. Indeed, using 6,914 whole bacterial genomes downloaded from a variety of publicly accessible repositories (via RepoPhlAn [https://bitbucket.org/nsegata/repophlan]), we observed that the percentage of shared 30-mers can be predicted from the percentage of shared 50-mers (Fig. 4b). A degree 3 polynomial was used (as it resulted in the lowest root mean square error [RMSE] and *R*^2^ values, which did not improve for higher-degree polynomials). Namely, we observed that for the polynomial *p*(*x*) = −.5141*x*.^3^ + 1.0932*x*.^2^ + 0.3824*x*, pckm_50_(*g*_*i*_,*g*_*j*_) ≈ *p*(pckm_30_(*g*_*i*_,*g*_*j*_)).

For *k*-mer lengths substantially shorter than 30, the behavior of pckm_*k*_ is more variable—for example, because of convergence of sequence composition between distantly related organisms. On the other hand, *k*-mers much larger than 50 are increasingly time consuming to compute and are likely to be more sensitive to sequencing error and other technical artifacts.

We can augment the matrices *A*^(*k*)^ with columns that represent hypothetical organisms which are related by different degrees to the reference organism. For a given organism with genome *g*_*i*_, if we wish to include a hypothetical organism *h* that is 90% similar to genome *g*_*i*_ in its 30-mers, we can round down each entry of the column vector pckm_30_(·,*g_i_*) to be no more than 0.90. Call this vector pckm_30_(·,*h*). The entries below 90% do not need to be changed, since we assume that the hypothetical organism has the same patterns of *k*-mer sharing to more distantly related “outgroup” taxa as to the reference organism.

We model the 50-mer similarity by setting pckm_50_(·,*h*) = *p*(pckm_30_(···,*g_i_*)) for the previously defined polynomial *p*. Adding these vectors to *A*^(30)^ and *A*^(50)^ effectively adds a hypothetical organism that has a common *k*-mer signature 90% similar to that of genome *g*_*i*_. We then repeat this procedure for all training genomes *g*_*i*_ and for similarities ranging from 90%, 80%,…,10% and append these columns to *A*^(30)^ and *A*^(50)^.

### (iii) Sample *k*-mer signature.

Given a metagenomic sample, we form two vectors, *y*^(30)^ and *y*^(50)^, consisting of the total counts in the sample of the 30-mers and 50-mers shared with the training organisms. In “Mathematical formulation” in Materials and Methods, we show that these vectors are linearly related to the organism abundances via the common *k*-mer matrices *A*^(30)^ and *A*^(50)^.

Note that in forming *y*^(*k*)^, we count the *k*-mers in the entire sample, not of the individual reads. This allows for a very computationally efficient approach: as the training genomes typically have low error, their *k*-mers can be efficiently stored in de Bruijn graphs (formed using Bcalm [22]). We can then query the bloom count filter formed from the sample in a highly parallel fashion.

### (iv) Sparsity-promoting optimization procedure.

After forming *y*^(*k*)^, we note that some of the entries *y*_*i*_^(*k*)^ may be nonzero due not to the presence of organism *i* in the sample but to the fact that there exists an organism *j* that shares portions of its genome with organism *i*. Since *A*_*i,j*_^(*k*)^ represents the “overlap” of these two organisms, we can deconvolute this linear mixture relationship by solving the equation *A*^(*k*)^*x* = *y*^(*k*)^ for *x*, the vector of organism abundances. However, after having augmented *A*^(*k*)^ with the hypothetical organisms, this system of equations is underdetermined (10 times more columns than rows). We can employ a sparsity-promoting optimization procedure to infer the most parsimonious *x* consistent with the equation *A*^(*k*)^*x* = *y*^(*k*)^ for *k* = 30, 50. This procedure, first introduced in reference 23 and proven correct in reference 24, is detailed in “Optimization procedure” in Materials and Methods.

### (v) Inferring taxonomy.

The abundances of the hypothetical organisms are then mapped back onto the taxonomy (for the output taxonomic profile) or the neighbor-joining tree formed from *A*^(*k*)^ (for the output strain variation figures), utilizing a least-common-ancestor approach detailed in “Inferring taxonomy” in Materials and Methods.

### Quantification of strain-level variation.

We demonstrate in two ways that the inclusion of the hypothetical organisms allows the inference of strain-level variation. First, we spike novel organisms into a mock metagenomic community and show that MetaPalette can accurately predict their presence. Second, we utilize a real metagenomic soil sample to give evidence for a novel strain that MetaPalette predicts.

### (i) HMP mock community.

**FIG 5:**
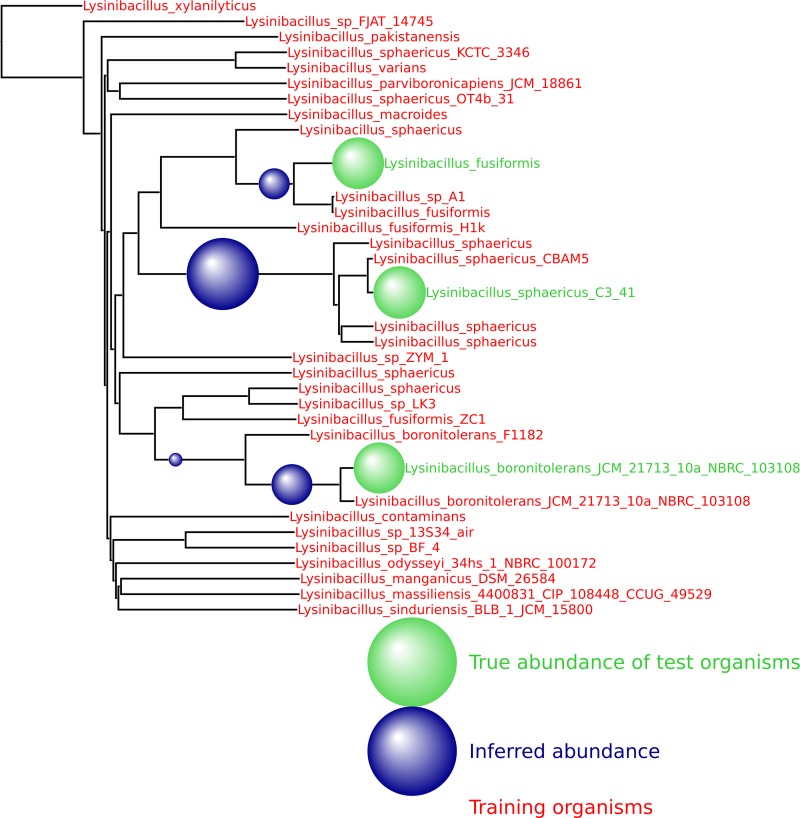
Result from training on 30 strains of *L. sphaericus* and testing on three novel strains. A total of 50,000 reads from the novel strains were spiked into the HMP mock even community. The training organisms are denoted with red font, and the names of the testing organisms are in green font.

We first formed the common *k*-mer matrices *A*^(*k*)^ using 31 strains of *Lysinibacillus sphaericus*. We then used Grinder (25) to simulate a data set consisting of two novel strains (not included in the training database). These reads were then spiked into the Human Microbiome Project (HMP) mock even community (an ~6.6 million-read metagenome consisting of 22 select organisms sampled using an Illumina GA-II sequencer; NCBI accession number SRR172902). The output of MetaPalette is shown in Fig. 5, demonstrating the ability of the method to correctly infer the presence of organisms absent from the training data.

**FIG 6:**
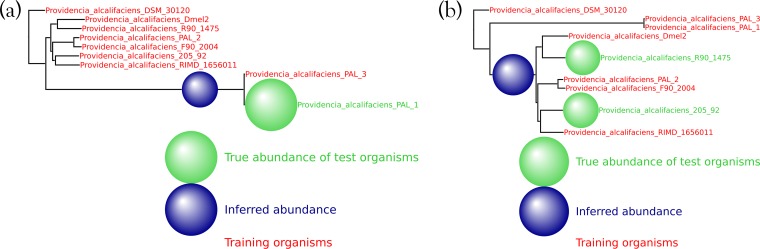
For each of the samples, a total of 50,000 reads from novel strains of *P. alcalifaciens* were spiked into the HMP mock even community. (a) Result from training on 8 strains and testing on 1 novel strain. (b) Result from training on 7 strains and testing on 2 novel strains.

Decreasing the number or changing the identity of the training organisms does not impede the method. As shown in Fig. 6, 50,000 simulated reads from the species *Providencia alcalifaciens* were again spiked into the HMP mock even community, and the inferred abundance was again placed optimally on the neighbor-joining tree. See Fig. 11 to 14 for a variety of such figures spanning all domains of life. These results provide evidence that MetaPalette can correctly infer the presence of organisms related to but absent from the training database.

### (ii) Metagenomic soil sample.

**FIG 7:**
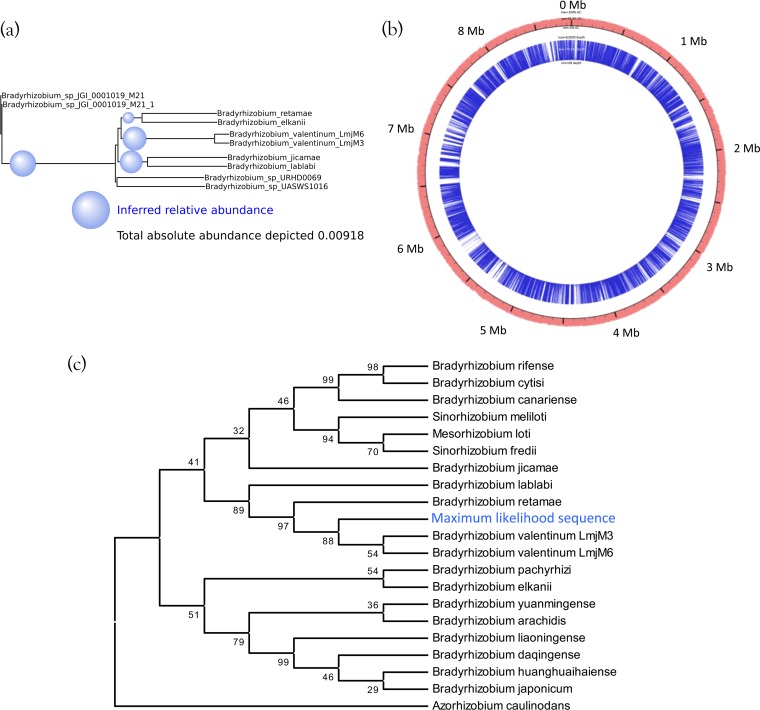
(a) Subtree of the MetaPalette output tree for the Iowa prairie metagenome using organisms from the genus *Bradyrhizobium*. (b) Depths of alignment for reads from the soil metagenome that aligned to *B. valentinum* LmjM3. The outer red ring shows the %GC for *B. valentinum* LmjM3, and the inner blue ring shows the alignment depth (truncated to 8,000× for ease of viewing). All contigs of the reference strain *B. valentinum* LmjM3 were concatenated in this figure. (c) Bootstrap consensus tree topology based on *nifH* for 20 organisms, along with the maximum-likelihood sequence obtained from aligning the soil metagenome to the *nifH* gene sequence of *B. valentinum* LmjM3. Bootstrap values (500 replicates) are shown next to the branches. Full details regarding the formation of the tree are given in “Sequence analysis details” in Materials and Methods.

To assess MetaPalette on a real metagenomic sample, we utilized the Iowa prairie metagenomic sample from reference 26 (corresponding to MG-RAST project accession number 6377). After running MetaPalette on a subset of these data (metagenome accession number 4539594.3), the taxonomic profile that was returned predicted the presence of the genus *Bradyrhizobium*. Generating the tree plot on a subset of this genus resulted in, among others, a prediction of a novel organism in the clade defined by strains of *Bradyrhizobium valentinum* (Fig. 7a). To verify this, we aligned the entire soil metagenome to the reference genome of the strain *B. valentinum* LmjM3 using Bowtie2 with –very-sensitive- local settings (27) and extracted the aligned reads. Interestingly, 0.29% of the reads aligned, while the MetaPalette-predicted abundance for this putative novel organism of interest was 0.33%. The depths of coverage of the extracted reads are pictured in Fig. 7b; the mean depth was 74.3×.

To assess the evolutionary relatedness of this predicted organism, we utilized the *B. valentinum* LmjM3 *nifH* gene sequence (NCBI accession number KF806461), which was used in the work reported in reference 28, along with other genes, to determine the taxonomy of *B. valentinum* LmjM3. Aligning the extracted reads to *nifH* resulted in a mean depth of coverage of 22×. We collapsed the aligned reads (via a majority vote) in regions of coverage of at least 22× and called this the maximum-likelihood sequence. We then performed a multiple-sequence alignment of this sequence along with the *nifH* sequences of 20 other organisms closely related to *B. valentinum*. The topology of the bootstrap consensus neighbor-joining tree is pictured in Fig. 7c and shows that the maximum-likelihood sequence is placed at the same location as was predicted by MetaPalette. While this is not enough evidence to unequivocally claim the existence of a novel strain in this sample, this gives support that MetaPalette correctly inferred the abundance and placement, as shown in Fig. 7a, of a potentially novel strain in the clade defined by strains of *B. valentinum*.

### Comparison to other metagenomic profiling methods.

To facilitate an objective comparison with other methods with minimal “author bias,” we utilized the same data and metrics used by other authors in a recent paper evaluating metagenomics methods (29). This allowed comparison to the following algorithms: CLARK (17), Kraken (16), OneCodex (30), LMAT (31), MG-RAST (32), MetaPhlAn (33), mOTU (14), Genometa (34), QIIME (35), EBI (36), MetaPhyler (15), MEGAN (37), taxator-tk (38), and GOTTCHA (39).

### (i) Training data.

Each of the methods was trained using the default recommended databases. We trained our method using 6,914 whole-genome sequences and assemblies obtained from various public repositories via RepoPhlAn (https://bitbucket.org/nsegata/repophlan). The training procedure for MetaPalette on these 6,914 organisms took a total of approximately 7 h on a 48-core server.

### (ii) Testing data.

The testing data consisted of 6 samples and are fully explained in reference 29 (in Methods), but we briefly summarize them here. Three replicates were formed from two different distributions of over 900 different genomes spanning the tree of life (including* Eukaryote* genomes). Included in each test sample were shuffled/randomized genomes (not meant to be assigned to any known taxon), as well as sequences from the genome of *Leptospira interrogans* that were evolved using Rose (40) to simulate novelty. Error profiles were based on those of 6 real soil metagenomic samples sequenced using an Illumina HiSeq 2000. Each of the resulting test samples contains between 27 and 37 million read pairs.

### (iii) Error metrics.

We utilized the same divergence error metric as was used in reference 29; that is, for *x*_*i*_, representing the true frequency of taxon *i* in the sample, and *x*_i_^*^, representing the predicted frequency of taxon *i* for a given method,

$$\text{divergence}=\underset{i}{\sum }\log_{2}\left(\frac{x_{i}^{*}}{x_{i}}\right)$$

where the summation is over those indices such that *X*_*i*_ > 0 and *x*_i_^*^ > 0. Since this error metric does not take into consideration the number of spurious assignments (that is, taxa predicted by a method to be in a sample but not actually present), we also use the number of false positives (FP) at a given taxonomic rank, as follows:

$$\text{FP}\;=\;\left|\left\{i\;:\;x_{i}^{*}>0\;\;\text{and}\;\;x_{i}\;=\;0\right\}\right|\cdot$$

### (iv) Comparison results.

**FIG 8:**
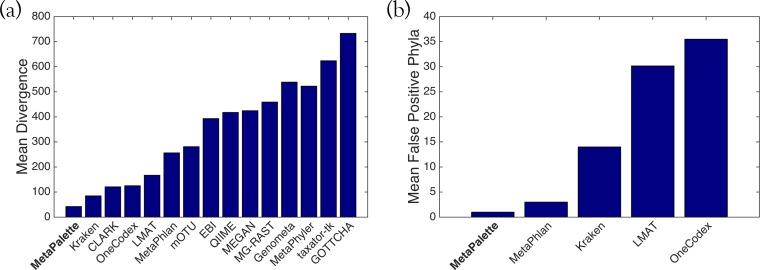
Plot of performance metrics for all metagenomics methods averaged over all test samples. Smaller values indicate better performance. (a) Divergence error metric at the genus level. (b) Number of false-positive phyla.

Each method was run using the default parameters. For each method, we averaged the divergence error metric over all the test samples at the genus level (Fig. 8a). Furthermore, we selected a number of the more accurate methods and averaged the number of false positives over all the test samples at the phylum level (Fig. 8b). The results in Fig. 8a and b clearly show the competitive nature of MetaPalette, as it has the lowest error using both metrics. However, when comparing to other methods, one should be careful of their intended use. For example, taxator-tk is intended to be used on an assembled metagenome (and here unassembled reads were used), and QIIME only uses the 16S rRNA sequences in a sample. Furthermore, most of these methods assign individual reads and then summarize them to obtain a taxonomic profile, while our method only profiles the entire sample and returns relative proportions of organisms.

**FIG 9:**
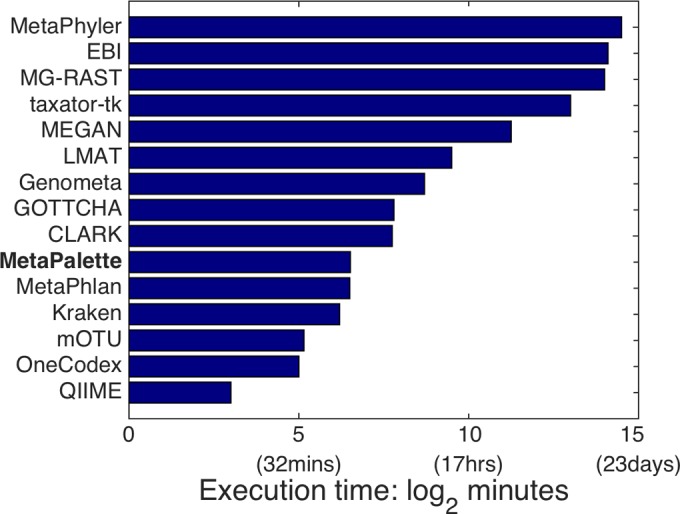
Mean execution time of each method averaged over all 6 test samples.

Figure 9 shows the execution time of each of the methods (on a log scale, obtained from reference 29), further showing the competitive nature of MetaPalette.

### Software and pretrained data. (i) Software.

The source code for MetaPalette, along with installation instructions and directions, is accessible at https://github.com/dkoslicki/MetaPalette. MetaPalette is written primarily in python and accepts input reads in uncompressed fasta or fastq format, as well as compressed fasta/fastq using bzip2 and gzip. For fastq input, optional parameters can be given to specify counting *k*-mers only above a certain quality score (Phred), thereby attenuating the negative impact of sequencing error in the correct inference of relative abundances. The output taxonomic profile is compliant with the Bioboxes profiling format, version 0.9, found at https://github.com/bioboxes/rfc/tree/master/data-format. Python scripts are also included to aid in downloading data, forming custom databases, and creating the appropriate taxonomy files.

To facilitate cross-platform usability, a Docker (41) container has been created and is accessible at: https://hub.docker.com/r/dkoslicki/metapalette, with an accompanying docker file at: https://github.com/dkoslicki/MetaPalette/blob/master/Docker/Dockerfile.

If users wish to use MetaPalette but lack computational resources, they may utilize the Galaxy (42–44) server located at: http://math-galaxy.cgrb.oregonstate.edu/.

A preliminary version of this software was submitted to Critical Assessment of Metagenomic Interpretation (CAMI: http://www.cami-challenge.org/) under the name CommonKmers. However, since significant changes have been made since that point, we strongly recommend using the current MetaPalette software instead.

### (ii) Pretrained data.

To decrease the computational burden, pretrained databases are accessible at http://files.cgrb.oregonstate.edu/Koslicki_Lab/MetaPalette. Databases and accompanying taxonomies have been included for *Archaea* (666 organisms; 1.7 GB uncompressed), *Bacteria* (15,147 organisms; 60 GB), *Eukaryota* (1,307 organisms; 41 GB), and viruses (4,798 organisms; 0.6 GB). All organisms were obtained via RepoPhlAn.

The 6,914-organism database used for the comparison to other profiling methods is accessible at http://files.cgrb.oregonstate.edu/Koslicki_Lab/MetaPalette (Comparison.tar.gz).

## DISCUSSION

We have described a fast, flexible, and accurate method for estimating the taxonomic composition of organisms which is based on reconstructing a *k*-mer-based profile of a sample. Each reference organism has a *k*-mer “palette,” and we fit the sample as a mixture of different palettes, both of the reference organisms and organisms absent from the training data at various degrees of relatedness to the training database. Our approach is in part inspired by the chromosome-painting method used to deduce fine-scale population structure in human genetics (45, 46), which is also based on mixture modeling of palettes. A particular advantage of MetaPalette over other metagenomic profiling methods is that MetaPalette provides an indication of how related the organisms in a given sample are to the closest matching organisms of the training database, whether they are within the same species or distantly related organisms from the same phyla.

Furthermore, the standard approach to summarizing composition information has been to place organisms at different taxonomic levels. We produce a standard taxonomic profile which we have shown to be more accurate than that produced by other methods. This fixed-rank approach is sensible at the genus level and above but omits fine-scale information. Hence, for branches of the tree of life that are well represented in the training database, we can also output a phylogenetic tree giving detailed information on how the sampled taxa relate to the organisms in the training database (Fig. 5 to 7; see also Fig. 11 to 14 in Materials and Methods).

For many applications, it is of interest to understand which individual reads belong to which organisms (1). A principled approach to this problem is to first estimate the overall composition of the sample, using MetaPalette or an equivalent, and then to assign individual reads conditional on the overall assignment. This represents a promising avenue for future methodological development.

## MATERIALS AND METHODS

### Mathematical formulation.

We include here all rigorous mathematical definitions of the quantities discussed in the main text. 

Given the alphabet 𝒜 = {A,C,T,G}, let 𝒜^*n*^ denote the set of all words *v* of length |v| = *n* on 𝒜, and let 𝒜* = ∪_*n*≥0_𝒜^*n*^ be the set of all finite words on 𝒜. Hence, words containing non-*ACTG* characters are ignored. Let *D* = {*g*_1_,···,*g_M_*} be a database of genomic sequences *g_j_* ∈ 𝒜* and let *S* = {*s*_1_,···,*s_N_*} be a set of sample sequences (the reads to be classified). For notational simplicity, assume that the read length is fixed; i.e., for all *t* = 1,…,*N* |*s_t_*| = *r*. Fix a *k*-mer size and endow 𝒜^*k*^ = {*v*_1_,···,*v*_4_*k*} with the lexicographic order. Let occ_v_(*w*) represent the number of occurrences (with overlap) of the subword *v* in the word *w*. That is, for *w*,*v* ∈ *A^n^*, let

(1) $${\text{occ}}_{v}\left(w\right)=\left|\left\{j:w_{j}w_{j+1}\cdot \cdot \cdot w_{j+|v|-1}=v\right\}\right|$$

For a fixed *k*-mer size and two genomes, *g*_*i*_ and *g*_*j*_, we calculate the number of *k*-mers in genome *j* common to both *g*_*i*_ and *g*_*j*_. That is, the (*i,j*)^th^ entry of the common *k*-mer training matrix *A*^(*k*)^ is as follows:

(2) $$A_{i,j}^{\left(k\right)}=\underset{\underset{w{\in \text{SW}}_{g_{i}}(k)\cap {\text{SW}}_{g_{j}}\left(k\right)}{}}{\sum }\frac{{\text{occ}}_{w}\left(g_{j}\right)}{\left|g_{j}\right|-k+1}$$

Refer to the entries of the common *k*-mer matrix as pckm_*k*_(*g_i_*,*g_j_*) = *A*_*i,j*_^(*k*)^. Let *s_i_* ⊂ *g_j_* denote the relationship that read *s*_*i*_ was derived from genome *g*_*j*_. We represent the taxonomic profile of the sample *S* by the probability vector *x* as follows:

(3) $$x_{j}=\frac{1}{N}\overset{N}{\underset{t=1}{\sum }}{𝟙}_{\left\{s_{t}{\subset g}_{j}\right\}}$$

where 𝟙 is the indicator function. Now let the measurement vector *y* be given by the probability vector

(4) $$y_{i}^{\left(k\right)}=\underset{\underset{w{\in \text{SW}}_{g_{i}}(k)\cap {\text{SW}}_{S}\left(k\right)}{}}{\sum }{\text{occ}}_{w}\left(S\right)/\underset{\underset{w{\in \text{SW}}_{S}\left(k\right)}{}}{\sum }{\text{occ}}_{w}\left(S\right)$$

We assume that the reads *s*_*t*_ are uniformly randomly selected from the genome *g*_*j*_. Then, for *w* ∈𝒜^*k*^, let ℙ(*w*|*g_j_*) be the probability that *k*-mer *w* is found in genome *g*_*j*_. Then, we have that the proportion of *k*-mers *w* in the sample is similar to the proportion of the appearance of *w* in the genome *g*_*j*_ when weighted by the relative abundance of the genome *g*_*i*_ in the sample, as follows:

(5) $$\frac{\sum_{t=1}^{N}{\text{occ}}_{w}\left(s_{t}\right)}{\sum_{{}_{w\in {\text{SW}}_{S}\left(k\right)}}{\text{occ}}_{w}(S)}\approx \frac{1}{N}\overset{M}{\underset{j=1}{\sum }}P(w|g_{j})\overset{N}{\underset{t=1}{\sum }}{𝟙}_{\left\{s_{t}{\;\subset \;g}_{j}\right\}}$$

(6) $$=\frac{1}{N}\overset{M}{\underset{j=1}{\sum }}\frac{{\text{occ}}_{g_{j}}\left(w\right)}{\left|g_{j}\right|-n+1}\overset{N}{\underset{t=1}{\sum }}{𝟙}_{\left\{s_{t\subset }g_{j}\right\}}$$

We then calculate

(7) $$y_{i}^{\left(k\right)}=\frac{1}{\sum_{w{\in \text{SW}}_{S}\left(k\right)}{\text{occ}}_{w}\left(S\right)}\underset{\underset{w\in {\text{SW}}_{g_{i}}\left(k\right)\cap {\text{SW}}_{S}\left(k\right)}{}}{\sum }{\text{occ}}_{w}\left(S\right)$$

(8) $$=\frac{1}{\sum_{{}_{w\in {\text{SW}}_{S}\left(k\right)}}{\text{occ}}_{w}\left(S\right)}\underset{\underset{w{\in \text{SW}}_{g_{i}}\left(k\right){\cap \text{SW}}_{S}\left(k\right)}{}}{\sum }\overset{N}{\underset{t=1}{\sum }}{\text{occ}}_{w}\left(s_{t}\right)$$

(9) ≈1N∑w∈SWgi(k)∩SWS(k)∑j=1Moccgj(w)|gj|−k+1∑t=1N𝟙{st⊂gj}

(10) $$=\frac{1}{N}\overset{M}{\underset{j=1}{\sum }}\underset{\underset{w\in {\text{SW}}_{g_{i}}(k)\cap {\text{SW}}_{g_{j}}\left(k\right){\cap \text{SW}}_{S}\left(k\right)}{}}{\sum }\frac{{\text{occ}}_{g_{j}}\left(w\right)}{\left|g_{j}\right|-k+1}\overset{N}{\underset{t=1}{\sum }}{𝟙}_{\left\{s_{t}{\subset g}_{j}\right\}}$$

(11) $$\approx \frac{1}{N}\overset{M}{\underset{j=1}{\sum }}\underset{w{\in \text{SW}}_{g_{i}}(k)\cap {\text{SW}}_{g_{j}}\left(k\right)}{\sum }\frac{{\text{occ}}_{g_{j}}\left(w\right)}{\left|g_{j}\right|-k+1}\overset{N}{\underset{t=1}{\sum }}{𝟙}_{\left\{s_{t\subset }g_{j}\right\}}$$

(12) $$=\overset{M}{\underset{j=1}{\sum }}A_{i,j}^{\left(k\right)}x_{j}={\left(A^{\left(k\right)}x\right)}_{i}$$

Equation 10 is justified since if *w*∉SW_*g_j_*_(*k*), then occ_*g_i_*_(*w*) = 0. For computational reasons, we make the assumption in equation 11 that SW*_g_i__*(*k*)∩SW*_g_j__*(*k*)∩SW_*S*_(*k*) = SW*_g_i__*(*k*)∩SW*_g_j__*(*k*). However, this assumption can be mitigated by adding hypothetical organisms (see “Hypothetical organisms” below). Our assumptions imply that

(13) $$A^{\left(k\right)}x{\;\approx y}^{\left(k\right)}$$

We will try to recover the vector *x* satisfying *x_j_* ≥ 0 for all *j* = 1,···,M from equation 13.

### Further improvements.

A few further improvements are possible, but not pursued here. Namely, we could use just the *k*-mers that are actually in the sample to form the training matrix. That is, use SW*_g_i__*(*k*)∩SW*_g_j__*(*k*)∩SW_*S*_(*k*) in the formation of *A*^(*k*)^, as follows:

$$A_{i,j}^{\left(k\right)}=\underset{}{}\underset{w{\in \text{SW}}_{g_{i}}\left(k\right){\cap \text{SW}}_{g_{j}}\left(k\right)\cap {\text{SW}}_{S}\left(k\right)}{\sum }\frac{{\text{occ}}_{w}\left(g_{j}\right)}{\left|g_{j}\right|-k+1}$$

The disadvantage of this is that the (slow) training step would need to be rerun for each sample.

For a second improvement, we could make the approximation in equation 5 more delicate by incorporating the coverage

(14) $$\frac{\sum_{t=1}^{N}{\text{occ}}_{w}\left(s_{t}\right)}{\sum_{{}_{w\in {\text{SW}}_{S}\left(k\right)}}{\text{occ}}_{w}\left(S\right)}\approx \frac{1}{N}\overset{M}{\underset{j=1}{\sum }}P(w{|g}_{j})\frac{\sum_{w{\in \text{SW}}_{g_{j}}(k){\cap \text{SW}}_{S}\left(k\right)}{\text{occ}}_{w}\left(g_{j}\right)}{{}_{\sum_{{w\in \text{SW}}_{g_{j}}\left(k\right)}}{\text{occ}}_{w}\left(g_{j}\right)}\overset{N}{\underset{t=1}{\sum }}{𝟙}_{\left\{s_{t}{\subset g}_{j}\right\}}$$

(15) $$=\frac{1}{N}\overset{M}{\underset{j=1}{\sum }}\frac{{\text{occ}}_{g_{j}}\left(w\right)}{\left|g_{j}\right|-n+1}\frac{\sum_{w{\in \text{SW}}_{g_{j}}(k){\cap \text{SW}}_{S}\left(k\right)}{\text{occ}}_{w}\left(g_{j}\right)}{\left|g_{j}\right|-k+1}\overset{N}{\underset{t=1}{\sum }}{𝟙}_{\{s_{t}\subset g_{j}\}}$$

So *A*^(*k*)^ would have the form:

(16) $$A_{i,j}^{\left(k\right)}=\frac{{}_{\sum_{w\in {\text{SW}}_{g_{j}}\left(k\right){\cap \text{SW}}_{S}(k)}occ_{w}(g_{j})}}{\left|g_{j}\right|-k+1}\underset{w{\in \text{SW}}_{g_{i}}\left(k\right){\cap \text{SW}}_{g_{j}}\left(k\right){\cap \text{SW}}_{S}\left(k\right)}{\sum }\frac{{\text{occ}}_{w}\left(g_{j}\right)}{|g_{j}|-k+1}$$

This effectively multiplies column *j* of *A*^(*k*)^ by the percent coverage of genome *j*. Finally, in equation 16, we could put a weighting factor that represents how unique a *k*-mer is to the genome in question. This would down-weight *k*-mers shared among many diverse genomes and up-weight those unique to certain strains/species/genera/etc.

### Hypothetical organisms.

To simulate an organism that is, say, 90% related to a database genome *g*_*i*_, we augment the common *k*-mer matrix *A*^(*k*)^ with a column derived by rounding down the entries of the column vector {*A*_*i,j*_^(*k*)^}_*i*=1,…,*M*_ that are above 90%. Two *k*-mer sizes are needed to form the hypothetical organism’s common *k*-mer matrices. For the first *k*-mer size, *k*_1_, we define *A*^(*k*_1_),*h*^ for a fixed number of hypothetical bins *h* ∈ {0.9,0.8,…,0.1}, where

(17) $$A_{i,j}^{\left(k_{1}\right),h}=\max\left[h,\underset{w{\in \text{SW}}_{g_{i}}(k_{1})\cap {\text{SW}}_{g_{j}}\left(k_{1}\right)}{\sum }\frac{{\text{occ}}_{w}\left(g_{j}\right)}{\left|g_{j}\right|-n+1}\right]$$

For the second *k*-mer size, *k*_2_, using the polynomial *p*(*x*) = −.5141*x*.^3^ + 1.0932*x*.^2^ + 0.3824*x*, we define

(18) $$A_{i,j}^{\left(k_{2}\right),h}=\max[h,p\;(\underset{w\in {\text{SW}}_{g_{i}}\left(k_{2}\right){\cap \text{SW}}_{g_{j}}\left(k_{2}\right)}{\sum }\frac{{\text{occ}}_{w}\left(g_{j}\right)}{|g_{j}|-n+1})]$$

Instead of thresholding, as we did here, one can imagine other scalings obtained from studying the relationship between a given taxonomy and the common *k*-mer matrix *A*^(*k*)^. In particular, to deal with differing rates of evolution in the tree of life, a fruitful area of future investigation would be to modify the polynomial *p* depending on the taxonomy of the organisms under consideration.

### Optimization procedure.

We choose two *k*-mer sizes to be *k*_1_ = 30 and *k*_1_ = 50, as this seems to give a good trade-off between reconstruction fidelity and computational performance. We then collect the common *k*-mer matrix and hypothetical matrices blockwise into the 2|*D*| × 10|*D*| size matrix as follows:

(19) $$A=\left[\begin{array}{cccc} A^{\left(30\right)}, & A^{\left(30\right),0.9}, & \cdot \cdot \cdot , & A^{\left(30\right),0.1}\\ A^{\left(50\right)}, & A^{\left(50\right),0.9}, & \cdot \cdot \cdot , & A^{\left(50\right),0.1} \end{array}\right]$$

Collect also the *k*-mer sample vectors *y*^(*k*)^:

(20) $$y=\left[\begin{array}{c} y^{\left(30\right)}\\ y^{\left(50\right)} \end{array}\right]$$

The problem at hand is then to reconstruct the phylogenetic profile *x* by solving the linear system

(21) Ax=y

Equation 21 is solved by using a sparsity-promoting optimization procedure motivated by techniques used in the compressive sensing literature. Sparsity is emphasized due to the inclusion of the hypothetical organisms, as well as the reasonable assumption that relatively few organisms from the database *D* are actually present in the given sample. We use a variant of nonnegative basis pursuit denoising which reduces to a nonnegative least-squares problem (24, 47). We aim to solve

$$x^{\text{∗}}=\underset{z}{\text{arg}\;\text{min}}||z||{}_{1}\;\;\text{subject}\;\text{to}\;Az=y,z\geq 0\;\;\;(\ell_{1}\text{-min})$$

This optimization procedure has the advantage of being transformed into a nonnegative least-squares problem. Indeed, as λ→∞, we can regularize (*ℓ*_1_-min) as

$$x^{*}=\text{arg}\;{\text{min}}_{z}\;||z||{}_{1}^{2}+\lambda^{2}||Az-y||{}_{2}^{2}\;\text{subject}\;\text{to}\;z\geq 0\;\;\;(\mathrm{NNREG})$$

This reduces to a nonnegative least-squares problem by defining

$$\overset{}{\overset{\sim }{A}}:=\left[\frac{1\cdot \cdot \cdot 1}{\lambda A}\right],\;\overset{}{\overset{\sim }{y}}:=\left[\frac{0}{\lambda y}\right]$$

So (NNREG) is equivalent to the nonnegative least-squares problem

$$x^{*}=\underset{z}{\arg\;\min\;\;}{\left\|\overset{\sim }{A}z-\overset{\sim }{y}\right\|}_{2}^{2}\;\;\mathrm{subject}\;to\;z\geq 0$$

This can be solved efficiently by using the LawsonHanson algorithm (48). We use the value λ = 200 throughout, as this value gives a good trade-off between sparsity and accuracy of fit of the *k*-mer counts.

### Inferring taxonomy.

Since the reconstructed vector *x* may have nonzero entries corresponding to a hypothetical bin, we need to develop a method to map from a hypothetical bin to a specific taxonomic rank. A naive approach would be to assign a fixed taxonomic rank to each hypothetical bin (call this the “fixed rank” method). For example, all nonzero entries of *x* corresponding to *A*^(*k*)^ would be assigned to the strain level, all nonzero entries of *x* corresponding to *A*^(*k*),0.9^ would be assigned to the species level, etc.

We take a more biologically informed approach: we take the least common ancestor (LCA) taxon between a hypothetical organism and a nearby organism in the database *D*: if *x_i_* > 0 corresponds to the hypothetical bin *h*, find an organism *g*_*j*_ such that |*A*_*i,j*_^(*k*)^ − *h*|<δ for some threshold δ. In the output taxonomic profile, we assign *x*_*i*_ to the lowest taxonomic rank common to the organisms with genomes *g*_*i*_ and *g*_*j*_. For the output strain variation figures, we assign the abundance *x*_*i*_ relative to the least common ancestor of *g*_*i*_ and *g*_*j*_ (above the LCA if *h* < *A*_*i,j*_^(*k*)^ and below the LCA if *h* > *A*_*i,j*_^(*k*)^).

For the output taxonomic profile, a hybrid of the fixed rank and LCA approaches can increase sensitivity or specificity. We thus include three options: the default option is the LCA approach, while the sensitive and specific options are various hybrids of the two methods.

### Sequence analysis details.

For the tree shown in Fig. 7c, the evolutionary history was inferred using the neighbor-joining method (49). The bootstrap consensus tree, inferred from 500 replicates, is taken to represent the evolutionary history of the taxa analyzed (50). Branches corresponding to partitions reproduced in less than 50% of the bootstrap replicates are collapsed. The percentages of replicate trees in which the associated taxa clustered together in the bootstrap test (500 replicates) are shown next to the branches. The analysis involved 21 nucleotide sequences. All positions with less than 95% site coverage were eliminated. That is, less than 5% alignment gaps, missing data, and ambiguous bases were allowed at any position. There were a total of 652 positions in the final data set. Evolutionary analyses were conducted in MEGA6 (51).

**FIG 10:**
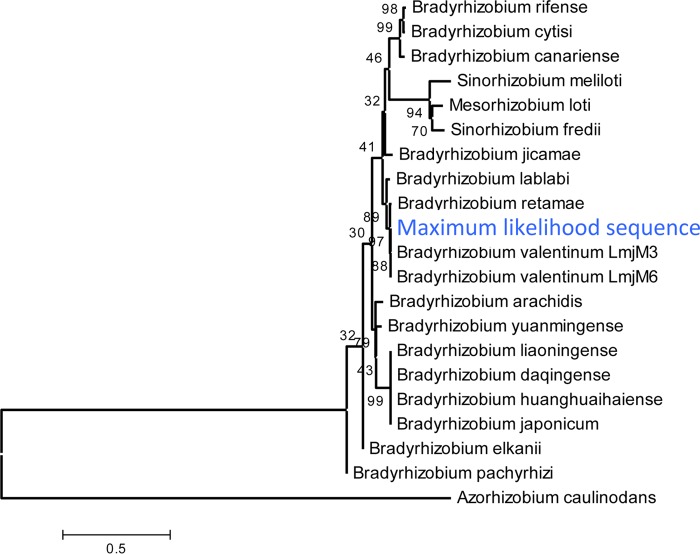
Neighbor-joining tree based on *nifH* for 20 organisms along with the maximum-likelihood sequence obtained from aligning the soil data to the gene sequence for *nifH* of *B. valentinum* LmjM3. Bootstrap values are shown next to the branches, and the bar indicates 0.5 nucleotide substitutions per site.

Figure 10 depicts a tree constructed using the same method as just described but with evolutionary distances computed using the maximum-composite-likelihood method (52). The unit is the number of base substitutions per site.

### Additional figures.

**FIG 11:**
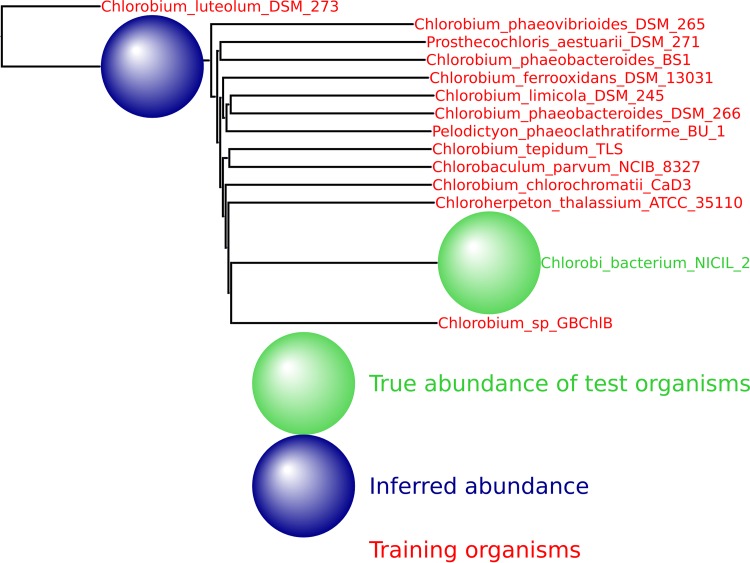
HMP spiking results for the bacterial phylum *Chlorobi* with 13 training organisms and 1 novel testing organism.

**FIG 12:**
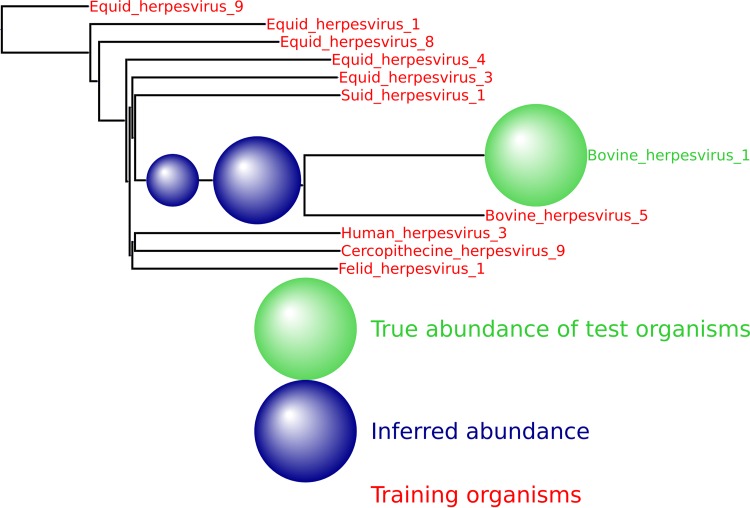
HMP spiking results for the viral genus *Varicellovirus* with 10 training organism and 1 novel testing organism. Only 5,000 simulated reads were spiked into the HMP sample.

**FIG 13:**
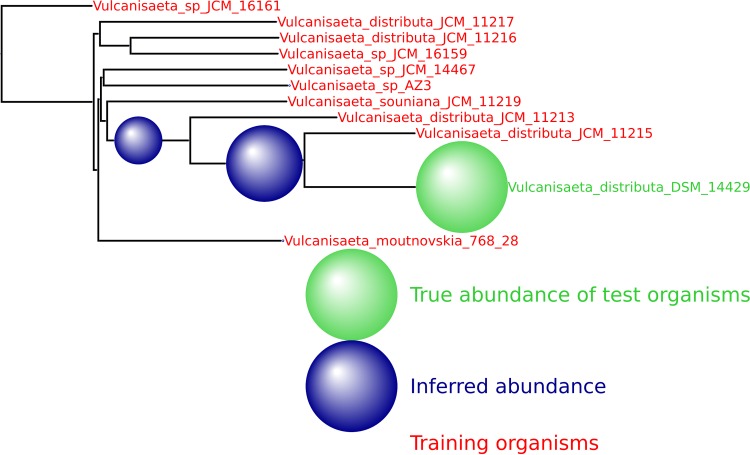
HMP spiking results for the archaeal genus *Vulcanisaeta* with 10 training organism and 1 novel testing organism. Only 5,000 simulated reads were spiked into the HMP sample.

**FIG 14:**
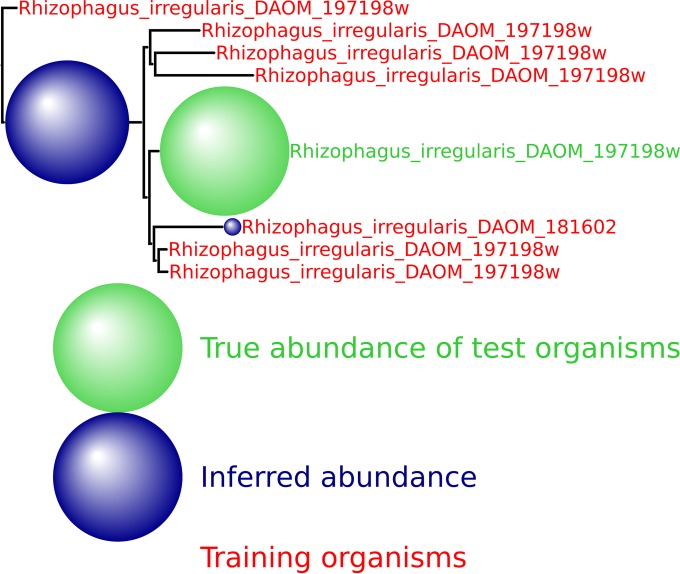
HMP spiking results for the *Eukaryota* genus *Rhizophagus* with 7 training organism and 1 novel testing organism.

We provide here a number of additional output figures from MetaPalette (Fig. 11 to 14) to demonstrate that the ability to correctly infer the presence of organisms related to but absent from the training database is not dependent on the particular kingdom/phyla/etc. used. Unless otherwise noted, a total of 50,000 simulated reads from the novel organisms were spiked into the HMP mock even community. Figures are included for *Bacteria*, *Archaea*, *Eukaryota*, and viruses.
